# Efficacy and safety of nanoparticle albumin-bound paclitaxel in advanced non-small cell lung cancer: A systematic review and meta-analysis of clinical trials and observational studies^☆^

**DOI:** 10.1016/j.heliyon.2023.e21903

**Published:** 2023-11-01

**Authors:** Nittiya Suwannasom, Netsai Sriaksorn, Chutamas Thepmalee, Chonthida Thephinlap, Patcharawan Tanamatayarat, Krissana Khoothiam, Hans Bäuemler, Nat Na-Ek

**Affiliations:** a Division of Biochemistry, School of Medical Sciences, University of Phayao, Phayao 56000, Thailand; b Division of Pharmaceutical Sciences, Department of Pharmaceutical Care, School of Pharmaceutical Sciences, University of Phayao, Phayao 56000, Thailand; c Unit of Excellence Technologies for Natural Products and Herbs, School of Pharmaceutical Sciences, University of Phayao, Phayao 56000, Thailand; d Division of Microbiology, School of Medical Sciences, University of Phayao, Phayao 56000, Thailand; e Institute of Transfusion Medicine, Center of Tumor Medicine, Charité-Universitätsmedizin Berlin, Charitéplatz 1, 10117 Berlin, Germany; f Division of Pharmacy Practice, Department of Pharmaceutical Care, School of Pharmaceutical Sciences, University of Phayao, Phayao 56000, Thailand; g Pharmacoepidemiology, Social and Administrative Pharmacy (P-SAP) Research Unit, School of Pharmaceutical Sciences, University of Phayao, Phayao 56000, Thailand

**Keywords:** Non-small cell lung cancer, Nanoparticle albumin-bound paclitaxel, Randomized controlled trial, Cohort study, Noncomparative study, Meta-analysis

## Abstract

**Background:**

The efficacy and safety of nanoparticle albumin-bound paclitaxel (nab-paclitaxel) in advanced non-small cell lung cancer (NSCLC) have yielded inconsistent findings.

**Materials and methods:**

We conducted a systematic review and meta-analysis, including comparative and noncomparative trials and cohort studies, to assess the efficacy and safety of nab-paclitaxel in advanced NSCLC. The search covered PubMed, CENTRAL, Scopus, and ClinicalTrials.gov until October 2022. Efficacy outcomes (OR, PR, progressive disease, OS, and PFS) and safety outcomes (neutropenia, leukopenia, thrombocytopenia, anemia, and sensory neuropathy) were analyzed.

**Results:**

Our meta-analysis included data from 35 studies (9 RCTs, 2 cohort studies, and 24 noncomparative studies). Nab-paclitaxel significantly improved OR rate (RR_RCT_ 1.35 [95% CI 1.19, 1.53], I^2^ = 36.6%; RR_cohort_ 1.67 [95% CI 1.30, 2.14], I^2^ = 4.3%) and PR rate (RR_RCT_ 1.34 [95% CI 1.18, 1.53], I^2^ = 38.8%; RR_cohort_ 1.59 [95% CI 1.22, 2.07], I^2^ = 19.4%) compared to the control group. It further demonstrated more pronounced benefits in squamous cell carcinoma and as a second-line treatment. Pooled evidence from the RCTs also indicated improved OS (HR 0.90 [95% CI 0.81, 0.99], I^2^ = 9.2%) and PFS (HR 0.84 [95% CI 0.76, 0.93], I^2^ = 14.5%) However, evidence on the reduction of adverse events with nab-paclitaxel treatment was insufficient, and biases in study selection and detection may have influenced the results.

**Conclusions:**

Nab-paclitaxel enhances OR, PR, PFS, and marginally improves OS in advanced NSCLC, particularly in patients with prior chemotherapy. Further research is needed to establish its safety advantages.

## Abbreviations

AEs: Adverse events
CENTRAL: Cochrane Central Register of Controlled Trials
CI: Confidence interval
CR: Complete response
CrEL: Cremophor EL
GRADE: Grading of Recommendations, Assessment, Development, and Evaluations
HR: Hazard ratio
IV: Inverse variance weighted
MINORS: Methodological Index for Non-randomized Studies
Nab-paclitaxel: Nanoparticle albumin-bound paclitaxel
Non-SCC: Non-squamous cell carcinoma
NOS: Newcastle-Ottawa score
NSCLC: Non-small cell lung cancer
OR: Overall response
OS: Overall survival
PFS: Progression-free survival
PR: Partial response
PRISMA: Preferred Reporting Items for Systematic reviews and Meta-analyses
PROSPERO: International Prospective Register of Systematic Reviews
RCT: Randomized controlled trial
RR: Relative risk
Sb-paclitaxel: Solvent-based paclitaxel
SCC: Squamous cell carcinoma
SD: Stable disease

## Introduction

Lung cancer is the second most commonly diagnosed cancer worldwide, with more than 2.2 million new cases estimated annually [1]. Among the lung cancer subtypes, non-small cell lung cancer (NSCLC) represents approximately 85% of cases. Unfortunately, a significant proportion (60%–70%) of NSCLC cases are diagnosed at an advanced stage, leading to a poor prognosis with a 5-year survival rate of only 16% [2].

Nanoparticle albumin-bound paclitaxel (nab-paclitaxel) is a novel formulation of paclitaxel that is free of solvents and offers potential advantages over conventional solvent-based paclitaxel (sb-paclitaxel) in terms of efficacy and safety. It reduces the risk of hypersensitivity reactions, eliminates the need for premedication, and allows for higher doses [3]. In contrast, conventional paclitaxel utilizes solvents like Cremophor EL (CrEL) and ethanol, associated with toxicities and potential reductions in drug efficacy [4]. Studies have shown that albumin-bound paclitaxel achieves a 10-fold increase in serum paclitaxel concentration and a 33% higher drug concentration in xenograft tumors than sb-paclitaxel [5,6]. Furthermore, the use of solvent-free nab-paclitaxel can potentially circumvent the toxicities associated with cremophor and reduce the incidence of allergic reactions [7]. In 2012, the Food and Drug Administration approved nab-paclitaxel for the treatment of NSCLC under the trade name Abraxane® [7].

Despite the approval of nab-paclitaxel for clinical use, its efficacy and safety profiles remain uncertain. Numerous clinical trials have investigated the efficacy and safety of nab-paclitaxel as monotherapy [[8], [9], [10], [11]] or in combination with different chemotherapeutic agent [[12], [13], [14], [15], [16]]. However, these studies have reported inconsistent findings and are often underpowered due to relatively small sample sizes. Furthermore, the validity of previous meta-analyses suggesting favorable efficacy and safety results for nab-paclitaxel in NSCLC may be limited by issues such as duplication of studies [17,18].

Therefore, we conducted a systematic review and meta-analysis of randomized controlled trials (RCTs), prospective cohort studies, and noncomparative studies to thoroughly evaluate the efficacy and safety of nab-paclitaxel in patients with advanced NSCLC. Our goal is to provide evidence-based guidance for treatment selection, with the objective of improving the survival rate in NSCLC.

## Materials and methods

This report follows the Preferred Reporting Items for Systematic reviews and Meta-analyses (PRISMA) statement 2020 (Supplemental Table 1) [19], and the study has been registered in the PROSPERO database (CRD42022364982).

### Eligibility criteria

Studies that investigated nab-paclitaxel alone or in combination with other chemotherapy agents, comparing it with regimens without nab-paclitaxel, for the treatment of NSCLC that met the following criteria were included: (1) clinical trials or longitudinal observational (cohort) studies involving patients aged at least 18 years old; (2) trials with at least one arm or observational studies that examined the efficacy and safety of nab-paclitaxel; and (3) had, for comparative studies, a control group that included solvent-based paclitaxel, placebo, or any other chemotherapy agents, except nab-paclitaxel itself.

To further clarify this criterion, when we subtract the intervention arm from the control arm, only nab-paclitaxel should remain in the comparison. For example, consider an RCT in which the intervention was carboplatin and nab-paclitaxel; if its control was docetaxel, then it would be excluded. However, if the control group in that study had been carboplatin, it would have been included in our analysis.

We excluded studies that (1) did not report at least one of the outcomes of interest; (2) had an unclear study design; and (3) were duplicates. Furthermore, in studies with more than two arms [15], only arms that compared nab-paclitaxel were included in the analysis.

### Search strategy

Two authors (NSu and NSri) searched electronic databases, including PubMed (1946 to present), Cochrane Central Register of Controlled Trials (CENTRAL; from inception to present), Scopus (2004 to present), and ClinicalTrials.gov (2000 to present). Additional studies were identified from the reference lists of relevant studies and reviews. The most recent search was conducted on October 11, 2022.

The search was not restricted to specific languages provided an English abstract was available. We used both Medical Subject Heading terms and relevant keywords to ensure comprehensive search results. The primary keywords used were “albumin-bound paclitaxel” AND “non-small cell lung cancer.” Further details on the search strategies used in this study can be found in Supplemental Appendix A.

#### Screening and selection process

Two authors (NSu and NSri) independently screened the titles and abstracts of the retrieved articles based on the eligibility criteria. Any disagreements were resolved by discussions with a third reviewer (NN). In rare cases in which duplicate studies presented similar risk of bias, the study with the larger sample size was included in the analysis. The screening process was performed using a Rayyan web application [20].

#### Data collection process

Two authors (NSu and NSri) independently performed data extraction using a predefined data extraction form. The form was validated through pilot tests on five randomly selected articles and was refined accordingly. The information extracted included: (1) research characteristics (e.g., first author, year of publication, study design, and characteristics of study populations; and treatment line); (2) details of the nab-paclitaxel regimen, including dosage and schedule; and (3) outcomes of interest (e.g., number of individuals experiencing the outcomes of interest, the total number in each group, and hazard ratio (HR) for survival outcomes with corresponding confidence intervals). In studies that reported results at multiple follow-up visits, we collected data from the visit with the longest follow-up duration.

#### Evaluation of the study risk of bias

To assess the risk of bias in trials with a control group (comparative trials) and single-arm (noncomparative) trials, we used the Cochrane risk of bias tool [21], and the Methodological index for non-randomized studies (MINORS) criteria [22], respectively. For comparative cohort studies, we used the Newcastle-Ottawa score (NOS) [23]. Detailed criteria for quality assessment using NOS can be found in the Supplemental Appendix B. Nevertheless, we did not assess the quality of noncomparative cohort studies, as there is no tool available for this purpose, and the implication from meta-analysis of noncomparative studies is currently limited.

To evaluate publication bias for each outcome of interest, we created a funnel plot displaying the effect size and corresponding inverse standard error for each study. If an asymmetrical shape was observed in the funnel plot, we additionally created a contour-enhanced funnel plot to examine the possibility of publication bias. Furthermore, we performed a nonparametric trim and fill analysis to assess the robustness of the findings by adding hypothetical studies that would make the funnel plot more symmetrical [24]. However, we did not perform statistical tests, such as Begg and Egger's tests, as they were likely to be statistically underpowered, particularly when fewer than 20 studies were included in the analysis for each result [25].

### Outcomes and effect measures

The primary outcome of our study is the overall response (OR), derived from complete and partial response (PR) cases. Secondary efficacy outcomes included complete response (CR), PR, stable disease (SD), progressive disease, progression-free survival (PFS), and overall survival (OS). Additionally, we pooled the disease control rate, which combines CR, PR, and SD. Regarding safety, we pooled adverse events (AEs) of grade 3 or above for the most commonly reported AEs, including anemia, neutropenia, thrombocytopenia, leukemia, and sensory neuropathy.

For comparative studies, most outcomes were pooled as relative risk (RR), except for PFS and OS, in which the HR was used as the pooled effect size. For noncomparative studies, the cumulative incidence was the pooled effect size. Corresponding 95% confidence intervals (95% CIs) were also reported for each pooled effect size.

### Statistical analysis

In the systematic review, we constructed a table comparing the characteristics and findings of the included studies without performing any further statistical analysis. For the meta-analysis, we primarily pooled the outcomes of interest using an inverse variance-weighted fixed-effect model, unless there was evidence of statistical heterogeneity. In such cases, we used the random-effects model with the DerSimonian-Laird estimator. For noncomparative studies, we pooled cumulative incidence using a single proportion approach with an Arcsine transformation [26]. To minimize methodological heterogeneity, the pooled effect sizes of observational studies, single-arm trials, and controlled trials were reported separately.

Statistical heterogeneity was assessed using Cochran's Q-statistic and I^2^. A P-value from the Q-statistic of <0.1 or an I^2^ value > 50% indicated evidence of statistical heterogeneity [25]. In such cases, we performed prespecified subgroup analyses to identify the source of heterogeneity, considering factors, such as types of control, histology, and whether nab-paclitaxel was administered as a first- or second-line regimen. Furthermore, to investigate the influence of individual studies, we performed a leave-one-out analysis for each outcome.

All analyses were performed using STATA version 16 MP (StataCorp LLC, College Station, Texas). Additional visualization, such as risk of bias assessment, was performed using Review Manager version 5.4, [27] and a GRADE evidence table was constructed using GRADEPro GDT [28].

## Results

### Study selection, characteristics, and risk of bias

**Fig. 1:**
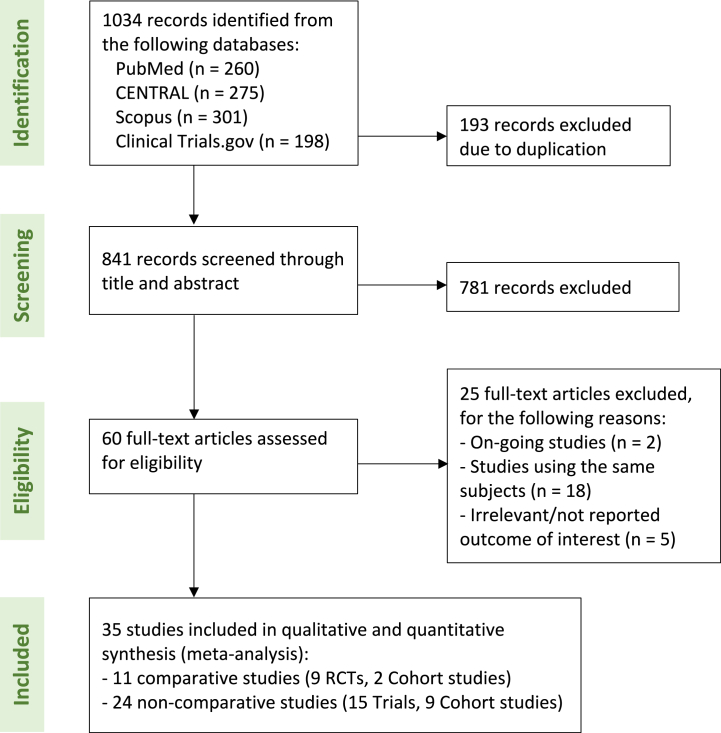
Study flow (PRISMA) diagram.

Initially, 1034 studies were identified from the four databases. Among these, a total of 35 studies were included in the meta-analysis, comprising nine RCTs with a sample size of 2858 and two cohort studies with a sample size of 471, and 24 noncomparative studies (15 trials with a sample size of 634 and nine cohort studies with a sample size of 442). The flow diagram of the study is presented in Fig. 1.

The characteristics of the included comparative and noncomparative studies are provided in Supplemental Tables 2 and 3, respectively. In summary, all the studies involved middle-to-older-aged adult patients (i.e., aged >50 years) with stage III or higher NSCLC, who were either treatment-naïve or who had failed previous chemotherapy. Treatment regimens varied and included monotherapy with nab-paclitaxel or combination regimens, particularly with carboplatin and cisplatin. The control groups in the RCTs included placebo; solvent-based paclitaxel; and other chemotherapeutic agents, such as pemetrexed, gemcitabine, and docetaxel.

Regarding the quality assessment of the included RCTs (Supplemental Figs. 1 and 2), there was an overall high risk of bias, particularly due to the potential detection bias resulting from the evaluation of the unblinded outcome. In the two comparative cohort studies, confounders were not adequately controlled, and one study lacked clear reporting of patient derivation and follow-up adequacy (Supplemental Table 4). Among the 15 noncomparative trials, 87% had high dropout rates and 53% showed potential bias in outcome assessment due to unblinded assessors (Supplemental Table 5).

## Results of comparative studies

**Fig. 2:**
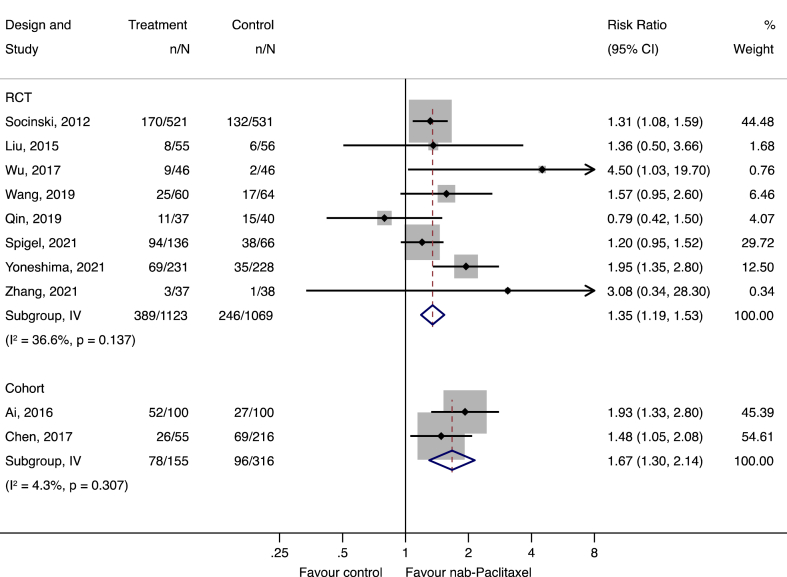
Forest plot of the overall response of nab-paclitaxel compared to the control. N**ote:** One randomized controlled trial [15] was dropped from the efficacy outcome analysis due to a data availability issue, but the results were included in the safety outcome analysis.

In terms of OR, a pooled analysis of eight RCTs and two cohort studies consistently indicated a statistically significant higher OR with nab-paclitaxel compared with the control group (pooled relative risks: RCTs 1.35 [95% CI 1.19, 1.53]; cohort studies 1.67 [95% CI 1.30, 2.14]) (Fig. 2).

**Fig. 3:**
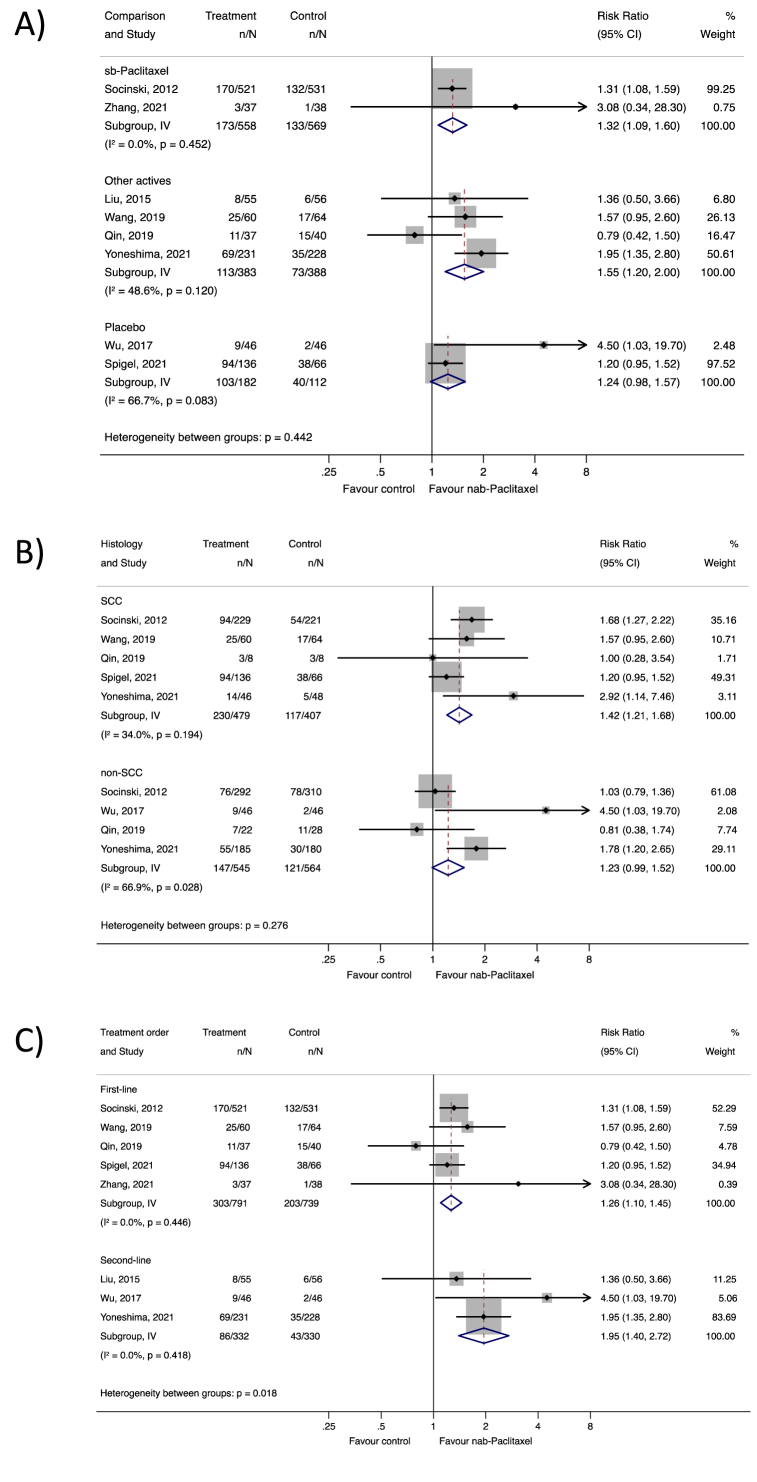
Subgroup analysis of the overall response according to the included RCTs by: A) comparison, B) histology, and C) by treatment order **Abbreviations:** CI, confidence interval; IV, inverse variance weighted; non-SCC, non-squamous cell carcinoma; RCTs, randomized controlled trial; sb-paclitaxel, solvent-based paclitaxel; SCC, squamous cell carcinoma.

The subgroup analysis of OR based on comparison types, histology subtypes, and first-or second-line use showed similar associations (Fig. 3). In particular, nab-paclitaxel had a stronger effect compared with other chemotherapeutic agents (RR 1.55 [95% CI 1.20, 2.00]) (Fig. 3A), among those with squamous cell carcinoma subtype (RR 1.42 [95% CI 1.21, 1.68]) (Fig. 3B), and as a second-line agent (RR 1.95 [95% CI 1.40, 2.72]) (Fig. 3C). Sensitivity analyses using a random-effects model confirmed these findings (Table 1), and subgroup analyses of PFS and OS outcomes were provided in Supplemental Figs. 3 and 4, respectively.

**Table 1:** Meta-analysis of efficacy outcomes of nab-paclitaxel according to comparative studies.

Efficacy outcomes	Design	N	Pooling effect size (95% CI)*		I^2^ , P-value for heterogeneity
			Fixed-effect model	Random-effects model	
Overall response	RCT	8	**1.35 (1.19, 1.53)**	**1.39 (1.14, 1.70)**	36.6%, 0.14
	Cohort	2	**1.67 (1.30, 2.14)**	**1.67 (1.29, 2.16)**	4.3%, 0.31
Disease control rate	RCT	7	1.01 (0.99, 1.04)	1.11 (0.98, 1.26)	78.3%, <0.001
	Cohort	2	**1.36 (1.23, 1.51)**	**1.33 (1.10, 1.60)**	63.8%, 0.096
Complete response	RCT	4	1.64 (0.40, 6.68)	1.64 (0.40, 6.68)	0.0%, 0.75
	Cohort	2	3.59 (0.94, 13.66)	3.59 (0.94, 13.66)	0.0%, 0.44
Partial response	RCT	7	**1.34 (1.18, 1.53)**	**1.37 (1.12, 1.67)**	38.8%, 0.13
	Cohort	2	**1.59 (1.22, 2.07)**	**1.59 (1.18, 2.15)**	19.4%, 0.26
Stable disease	RCT	7	0.94 (0.83, 1.06)	0.95 (0.79, 1.14)	43.2%, 0.10
	Cohort	2	1.04 (0.79, 1.37)	0.96 (0.44, 2.07)	86.6%, 0.006
Progressive disease	RCT	7	**0.76 (0.64, 0.90)**	0.76 (0.58, 1.00)	49.4%, 0.065
	Cohort	2	**0.56 (0.39, 0.82)**	0.49 (0.23, 1.05)	67.5%, 0.079
Overall survival	RCT	7	**0.90 (0.81, 0.99)**	0.90 (0.80, 1.00)	9.2%, 0.36
	Cohort	0	NA	NA	NA
Progression-free survival	RCT	7	**0.84 (0.76, 0.93)**	**0.84 (0.75, 0.94)**	14.5%, 0.32
	Cohort	0	NA	NA	NA

**Note:** *Risk ratio, except for overall survival and progression-free survival in which the effect size is the hazard ratio, an embolden figure represents a statistically significant value. A RCT [15] was dropped from the efficacy outcome analysis due to a data availability problem, but its results were included in the analysis of safety outcomes.

In addition to the OR, nab-paclitaxel demonstrated benefits in improving PR rate (RR_RCT_ 1.34 [95% CI 1.18, 1.53]; RR_cohort_ 1.59 [95% CI 1.22, 2.07]), OS (HR 0.90 [95% CI 0.81, 0.99]), and PFS (HR 0.84 [95% CI 0.76, 0.93]) compared with the control group (Table 1).

Most sensitivity analyses supported these associations, except for OS, which showed attenuated results with a random-effects model (Table 1). No evidence of publication bias or influential studies was found (Supplemental Figs. 5–10). Collectively, GRADE evidence (Table 2) indicated moderate certainty of the benefits of nab-paclitaxel on OR, PR, and PFS, while the impact on OS was of low certainty.

**Table 2:** GRADE evidence of efficacy of nab-paclitaxel according to included RCTs.

Outcomes	Anticipated absolute effects* (95% CI)		Relative effect (95% CI)	№ of participants (studies)	Certainty of the evidence (GRADE)	Comments
	Risk with sb-paclitaxel	Risk with nab-paclitaxel				
Overall response (OR) assessed with: RECIST criteria follow-up: range 24–96 months	230 per 1000	311 per 1000 (274, 352)	RR 1.35 (1.19, 1.53)	2192 (8 RCTs)	⨁⨁⨁◯ Moderate^a^^,^^b^^,^^c^	Nab-paclitaxel improved the overall response rate by 35% (19%, 53%) compared with sb-paclitaxel.
Partial response (PR) assessed with: RECIST criteria follow-up: range 24–96 months	236 per 1000	316 per 1000 (278, 361)	RR 1.34 (1.18, 1.53)	2117 (7 RCTs)	⨁⨁⨁◯ Moderate^a^^,^^b^^,^^c^	Nab-paclitaxel improved the partial response rate by 34% (18%, 53%) compared with sb-paclitaxel.
Progression-free survival (PFS) follow-up: range 24–96 months	Not calculated		HR 0.84 (0.76, 0.93)	2117 (7 RCTs)	⨁⨁⨁◯ Moderate^a^^,^^b^^,^^c^	Patients who received nab-paclitaxel had a 16% (7%, 24%) lower risk of disease progression or death (or 19% increase in progression-free survival time) than individuals who received sb-paclitaxel.
Overall survival (OS) follow-up: range 24–96 months	Not calculated		HR 0.90 (0.81, 0.99)	2117 (7 RCTs)	⨁⨁◯◯ Low^a^^,^^b^^,^^c^^,^^d^	Patients who received nab-paclitaxel had a 10% (1%, 19%) lower risk of death (or 11% increase in survival time) than those who received sb-paclitaxel.
*The risk in the intervention group (and its 95% confidence interval) is based on the assumed risk in the comparison group and the relative effect of the intervention (and its 95% CI). CI: confidence interval; HR: hazard Ratio; RR: risk ratio						
**GRADE Working Group grades of evidence** **High certainty:** we are very confident that the true effect lies close to that of the estimate of the effect. **Moderate certainty:** we are moderately confident in the effect estimate: the true effect is likely to be close to the estimate of the effect, but there is a possibility that it is substantially different. **Low certainty:** our confidence in the effect estimate is limited: The true effect may be substantially different from the estimate of the effect. **Very low certainty:** we have very little confidence in the effect estimate: the true effect is likely to be substantially different from the estimate of effect.						

Explanations.

a Potential selection bias due to unclear random sequence generation or allocation concealment.

b Potential detection bias due to unblinded outcome assessment.

c Potential bias due to unbalanced baseline characteristics between groups.

d Inconsistency findings between fixed and random-effects models (although a small degree of statistical heterogeneity was detected).

In contrast to the efficacy outcomes, the safety analysis of nab-paclitaxel did not demonstrate significant benefits compared with the control group (Table 3). Due to inconsistent results in the sensitivity analyses and the high degree of statistical heterogeneity, the validity of the safety findings could not be ensured in our study.

**Table 3:** Meta-analysis of safety outcomes (i.e., adverse events grade ≥3) of nab-paclitaxel based on comparative studies.

Safety outcomes	Design	N	Pooling risk ratio (95% CI)*		I^2^ , P-value for heterogeneity
			Fixed-effect model	Random-effects model	
Hematologic outcomes					
Neutropenia	RCT	7	**0.78 (0.69, 0.88)**	1.19 (0.66, 2.16)	93.7%, <0.001
Leukopenia	RCT	5	**0.47 (0.38, 0.58)**	1.33 (0.47, 3.75)	85.6%, <0.001
Thrombocytopenia	RCT	7	1.15 (0.86, 1.54)	1.23 (0.75, 2.03)	52.1%, 0.051
	Cohort	2	**0.63 (0.42, 0.94)**	1.18 (0.17, 8.16)	91.4%, 0.001
Anemia	RCT	7	**1.49 (1.17, 1.89)**	1.42 (0.83, 2.41)	71.7%, 0.002
**Non hematologic outcomes**					
Sensory neuropathy	RCT	5	2.13 (0.99, 4.57)	3.36 (0.57, 19.95)	78.6%, 0.001

**Note:** *The pooled risk ratio of experiencing a specific adverse event in the nab-paclitaxel group, compared to the control group, where a value > 1 and < 1 indicates an increased (favouring the control group) and decreased (favouring the nab-paclitaxel group) risk of the adverse event, respectively. An emboldened figure represents a statistically significant value (P-value <0.05).

### Results of noncomparative studies

In terms of efficacy, noncomparative studies (Supplemental Table 6) demonstrated similar cumulative incidence rates for OR (22%), disease control (64%), or death (2%). However, the moderate-to-substantial degree of heterogeneity in these studies suggests that the efficacy results should be interpreted with caution.

Regarding safety, the most frequently reported hematological adverse event was neutropenia, with a cumulative incidence of 25% (95% CI 16%, 34%, I^2^ = 83.2%) in the included trials (Supplemental Table 7). Nonhematological AEs, such as sensory neuropathy and arthralgia, were reported in small proportions in both trials and cohort studies. It is important to note that the results from noncomparative studies were descriptive in nature, and that causal or associative inferences could not be made.

## Discussion

### Summary of key findings

From the findings of this systematic review and meta-analysis of 35 studies, including 4405 patients with advanced NSCLC, we are moderately certain that nab-paclitaxel can improve OR, PR, and PFS. In terms of OR, the effect of nab-paclitaxel is particularly evident compared with other chemotherapeutic agents, especially in the subtype of squamous cell carcinoma and in patients who received it as second-line treatment. However, we have low certainty about the impact of nab-paclitaxel on OS. Conversely, the impact of nab-paclitaxel on safety outcomes is inconclusive. The high degree of statistical heterogeneity, inconsistent findings in sensitivity analyses, and imprecise confidence intervals suggest that further investigation of the safety of nab-paclitaxel is still required [29].

### Comparing with previous studies

The current analysis contributes to recent meta-analyses assessing the efficacy and safety of nab-paclitaxel in NSCLC treatment [17,18]. Both studies showed an improved OR rate, PFS, and marginally prolonged OS with nab-paclitaxel, similar to our findings. However, their results were duplicated by including studies [[30], [31], [32], [33]] with the same population as Socinski et al. [12] The meta-analysis by Han et al. included a different nano system, polymeric micellar paclitaxel, which introduced clinical heterogeneity and may the undermine internal validity and generalizability [34].

Our safety findings differ from those of previous studies. We did not find an association between nab-paclitaxel and the safety profile, while previous studies showed a decreased risk of neutropenia, alopecia, sensory neuropathy, and arthralgia, but an increased risk of thrombocytopenia, anemia, and nausea in the nab-paclitaxel group [17,18]. This discrepancy may be due to duplication issues. For instance, the risk ratio (95% CI) for neutropenia in Socinski et al. was 0.83 (95% CI 0.57, 1.19) in the nab-paclitaxel group compared with the control [12]. If the cohort was counted multiple times, the results would likely be biased toward nab-paclitaxel. In particular, our fixed-effect models of included RCTs also showed a significantly lower risk of neutropenia and leukopenia, but a higher risk of thrombocytopenia, anemia, and sensory neuropathy in the nab-paclitaxel group. However, due to substantial heterogeneity, we utilized a random-effects model, attenuating the results toward the null.

In the non-comparative studies, the most frequently reported adverse events were neutropenia (25%) and leukopenia (10%). This is consistent with the results of a meta-analysis conducted by Tan et al., which found that the most frequently observed adverse events were neutropenia (22.1%), leukopenia (14.8%), anemia (8.0%), and thrombocytopenia (8.0%) [18].

In our study, however, the nab-paclitaxel group had a lower incidence of thrombocytopenia (3%) and anemia (0%). These differences may be attributed to variations in the calculation methods. Specifically, previous work simply added the number of participants experiencing adverse events divided by the total number of participants from all trials. In contrast, our results were pooled using the meta-analysis approach for combining a single proportion, which accounts for both within- and between-study variability. Therefore, our safety findings are more reliable.

### Strengths and limitations

To our knowledge, this is the most comprehensive and up-to-date analysis reported till date. We performed sensitivity analyses and used the GRADE strategy to ensure the robustness and applicability of our findings in clinical practice. In addition, we carefully selected and included trials that were not duplicated to strengthen the internal validity of our findings. Although our study may not introduce significantly innovative insights compared to previous meta-analyses related to nab-paclitaxel [17,18], it does provide more reliable findings, enhancing its usefulness for clinical practice.

However, there were several limitations to our study that should be considered. First, most participants in the included RCTs were men, which may limit the generalizability of our findings, as sex can influence the prognosis in lung cancer [35]. Secondly, our analysis included studies with a wide range of nab-paclitaxel dosage regimens, making it challenging to determine the optimal dose for clinical use based on our results.

Furthermore, the reliability of our findings depends on the quality of the included studies, particularly the RCTs. It is concerning that most of the included RCTs did not employ blinded outcome assessors, which introduces detection bias and potentially overestimates the intervention effect [36,37]. This is particularly problematic for outcomes based on human judgment, such as PFS and the OR rate [38].

A notable example is a landmark study on cetuximab in patients with advanced NSCLC, in which unblinded investigators reported statistically significant improvements in OR rate and PFS in the cetuximab arm, while independent radiological review committee evaluations indicated null results [39]. Therefore, caution should be exercised when interpreting the efficacy observed in our study, as it may be biased in favor of nab-paclitaxel. However, the marginal benefit achieved by nab-paclitaxel in OS, which was less susceptible to detection bias [40], may still be relevant in patients with advanced NSCLC.

## Implications

Our meta-analysis suggests that nab-paclitaxel may provide benefits to patients with advanced NSCLC, supporting the decision-making process leading to drug approval and expanding its clinical use. However, the lack of blinded outcome assessors highlights the need for additional well-designed trials.

Furthermore, subgroup analysis indicates a potential benefit of nab-paclitaxel specifically for patients with squamous cell histology and those who have undergone previous chemotherapy. However, these findings should be considered preliminary and further studies are required for validation. Specifically, additional research is necessary to confirm the superior OR rate in advanced NSCLC with squamous cell histology and to establish the effectiveness of nab-paclitaxel as a second-line treatment.

Due to the uncertainties surrounding safety outcomes, it is essential to conduct well-executed studies, including post-marketing vigilance studies, to thoroughly evaluate the safety profile and benefits of nab-paclitaxel. Notably, almost all of the included RCTs examined the efficacy and safety of nab-paclitaxel at a dose of 100 mg/m^2^ or higher, which could potentially be reduced to 70 mg/m^2^, as suggested by one RCT. This reduction may lead to a decrease in toxicity and treatment costs while still achieving similar efficacy [41]. Future clinical trials are required to confirm the efficacy and safety of a lower optimal dose of nab-paclitaxel before its implementation in clinical practice.

## Conclusions

Our systematic review and meta-analysis of 35 studies, involving 4405 patients with advanced NSCLC, suggests that nab-paclitaxel improved OR, PR, and PFS. In particular, nab-paclitaxel demonstrated greater effectiveness compared with other chemotherapeutic agents in terms of OR, particularly in squamous cell carcinoma and as a second-line treatment. However, the positive impact of nab-paclitaxel on OS is modest, while its effect on safety outcomes remains inconclusive. Given the high heterogeneity, inconsistent findings, and imprecise confidence intervals, further investigation is warranted into the safety of nab-paclitaxel.

## Ethics declarations

Review and/or approval by an ethics committee was not needed for this study because it is a systematic review and meta-analysis that utilized published summary data. Informed consent was not required for this study because the authors neither had access to individual patient-level data nor directly approached individual patients.

Funding and role of funding source.

This work was supported by the 10.13039/501100017170Thailand Science Research and Innovation Fund and the 10.13039/501100011093University of Phayao (Grant No. UoE62010). Nevertheless, the funding body did not participate in study design; in the collection, analysis and interpretation of data; in the writing of the report; and in the decision to submit the article for publication.

## Data availability statement

The data associated with this study have not been deposited into any publicly available repository. However, the data will be made available upon request.

## CRediT authorship contribution statement

**Nittiya Suwannasom:** Writing – original draft, Visualization, Validation, Project administration, Methodology, Investigation, Formal analysis, Data curation, Conceptualization, Asst Prof. **Netsai Sriaksorn:** Investigation, Formal analysis, Data curation, Asst Prof. **Chutamas Thepmalee:** Writing – review & editing, Methodology, Investigation, Conceptualization, Asst Prof. **Chonthida Thephinlap:** Writing – review & editing, Methodology, Conceptualization, Asst Prof. **Patcharawan Tanamatayarat:** Writing – review & editing, Methodology, Funding acquisition, Conceptualization, Asst Prof. **Krissana Khoothiam:** Writing – review & editing, Validation, Conceptualization, Prof. **Hans Bauemler:** Writing – review & editing, Validation, Supervision, Conceptualization. **Nat Na-Ek:** Writing – review & editing, Writing – original draft, Visualization, Validation, Supervision, Methodology, Investigation, Formal analysis, Data curation, Conceptualization.

## Declaration of Generative AI and AI-assisted technologies in the writing process

During the preparation of this work the authors used ChatGPT, an AI language model developed by OpenAI, to improve readability and language. After using this tool, the authors reviewed and edited the content as needed and take full responsibility for the content of the publication.

## Declaration of competing interest

The authors declare the following financial interests/personal relationships which may be considered as potential competing interests.

## Footnotes

Appendix A Supplementary data to this article can be found online at https://doi.org/10.1016/j.heliyon.2023.e21903.
