# Supplementary material for: Efficacy and safety of nanoparticle albumin-bound paclitaxel in advanced non-small cell lung cancer: A systematic review and meta-analysis of clinical trials and observational studies
Source: Heliyon. 2023 Nov 1;9(11):e21903. doi: 10.1016/j.heliyon.2023.e21903 (PMC10660490; doi:10.1016/j.heliyon.2023.e21903)
Supplement: Supplementary file 1 [file mmc1.docx]

**Supplementary appendices**

This is supplement to the article entitled: “Efficacy and safety of nanoparticle albumin-bound paclitaxel in advanced non-small cell lung cancer: a systematic review and meta-analysis of clinical trials and observational studies”

[**Supplementary Appendix A:** Search strategies 4](#_Toc136996274)

[**Supplementary Appendix B:** NOS key criteria for assessment 9](#_Toc136996275)

[**Supplementary Appendix C**: eReferences 24](#_Toc136996276)

**Supplementary Tables**

[**Supplementary Table 1** PRISMA checklist 3](#_Toc135293719)

[**Supplementary Table 2** Characteristics of included comparative studies 5](#_Toc135293720)

[**Supplementary Table 3** Characteristics of included non-comparative studies 6](#_Toc135293721)

[**Supplementary Table 4** Quality assessment of included comparative cohort studies 10](#_Toc135293722)

[**Supplementary Table 5** Risk of bias of included non-comparative trials 11](#_Toc135293723)

[**Supplementary Table 6** Cumulative incidence of efficacy outcomes according to non-comparative studies 14](#_Toc135293724)

[**Supplementary Table 7** Cumulative incidence of safety outcomes according to non-comparative studies 15](#_Toc135293725)

**Supplementary Figures**

[**Supplementary Figure 1** Risk of bias of individual RCT 8](#_Toc135293726)

[**Supplementary Figure 2** Summary risk of bias of included RCTs 8](#_Toc135293727)

[**Supplementary Figure 3** Subgroup analysis of progress-free survival (PFS) according to included RCTs 12](#_Toc135293728)

[**Supplementary Figure 4** Subgroup analysis of overall survival (OS) according to included RCTs 13](#_Toc135293729)

[**Supplementary Figure 5** Contour-enhanced funnel plots of efficacy outcomes according to included RCTs 17](#_Toc135293730)

[**Supplementary Figure 6** Funnel plots of efficacy outcomes according to non-comparative studies 18](#_Toc135293731)

[**Supplementary Figure 7** Contour-enhanced funnel plots of safety outcomes according to included RCTs 19](#_Toc135293732)

[**Supplementary Figure 8** Funnel plots of safety outcomes according to non-comparative studies 21](#_Toc135293733)

[**Supplementary Figure 9** Leave-one-out analysis of efficacy outcomes according to comparative studies 22](#_Toc135293734)

[**Supplementary Figure 10** Leave-one-out analysis of safety outcomes according to comparative studies 23](#_Toc135293735)

**Supplementary Table 1** PRISMA checklist

| **Section/topic** | **Item No.** | **Checklist item** | **Reported on Page No.** |
| --- | --- | --- | --- |
| **Title** |  |  |  |
| Title | 1 | Identify the report as a systematic review, meta-analysis, or both | 1 |
| **Abstract** |  |  |  |
| Structured summary | 2 | Provide a structured summary including, as applicable, background, objectives, data sources, study eligibility criteria, participants, interventions, study appraisal and synthesis methods, results, limitations, conclusions and implications of key findings, systematic review registration number | 3-4 |
| **Introduction** |  |  |  |
| Rationale | 3 | Describe the rationale for the review in the context of what is already known | 7-8 |
| Objectives | 4 | Provide an explicit statement of questions being addressed with reference to participants, interventions, comparisons, outcomes, and study design (PICOS) | 8 |
| **Methods** |  |  |  |
| Protocol and registration | 5 | Indicate if a review protocol exists, if and where it can be accessed (such as web address), and, if available, provide registration information including registration number | 8, PROSPERO database (CRD42022364982) |
| Eligibility criteria | 6 | Specify study characteristics (such as PICOS, length of follow-up) and report characteristics (such as years considered, language, publication status) used as criteria for eligibility, giving rationale | 9 |
| Information sources | 7 | Describe all information sources (such as databases with dates of coverage, contact with study authors to identify additional studies) in the search and date last searched | 9-10 |
| Search | 8 | Present full electronic search strategy for at least one database, including any limits used, such that it could be repeated | Supplementary Appendix A |
| Study selection | 9 | State the process for selecting studies (that is, screening, eligibility, included in systematic review, and, if applicable, included in the meta-analysis) | 10 |
| Data collection process | 10 | Describe method of data extraction from reports (such as piloted forms, independently, in duplicate) and any processes for obtaining and confirming data from investigators | 10-11 |
| Data items | 11 | List and define all variables for which data were sought (such as PICOS, funding sources) and any assumptions and simplifications made | 10-11,  Supplementary Table 2-3 |
| Risk of bias in individual studies | 12 | Describe methods used for assessing risk of bias of individual studies (including specification of whether this was done at the study or outcome level), and how this information is to be used in any data synthesis | 11-12,  Supplementary Appendix A |
| Summary measures | 13 | State the principal summary measures (such as risk ratio, difference in means). | 12-13 |
| Synthesis of results | 14 | Describe the methods of handling data and combining results of studies, if done, including measures of consistency (such as I^2^) for each meta-analysis | 13-14 |
| Risk of bias across studies | 15 | Specify any assessment of risk of bias that may affect the cumulative evidence (such as publication bias, selective reporting within studies) | 12 |
| Additional analyses | 16 | Describe methods of additional analyses (such as sensitivity or subgroup analyses, meta-regression), if done, indicating which were pre-specified | 13-14 |
| **Results** |  |  |  |
| Study selection | 17 | Give numbers of studies screened, assessed for eligibility, and included in the review, with reasons for exclusions at each stage, ideally with a flow diagram | Figure 1 |
| Study characteristics | 18 | For each study, present characteristics for which data were extracted (such as study size, PICOS, follow-up period) and provide the citations | Supplementary Table 2-3 |
| Risk of bias within studies | 19 | Present data on risk of bias of each study and, if available, any outcome-level assessment (see item 12). | Supplementary Figure 1-2,  Supplementary Table 4-5 |
| Results of individual studies | 20 | For all outcomes considered (benefits or harms), present for each study (a) simple summary data for each intervention group and (b) effect estimates and confidence intervals, ideally with a forest plot | Figure 2, Table 1, 3, Supplementary Table 6-7 |
| Synthesis of results | 21 | Present results of each meta-analysis done, including confidence intervals and measures of consistency | Figure 2, Table 1-3, Supplementary Table 6-7 |
| Risk of bias across studies | 22 | Present results of any assessment of risk of bias across studies (see item 15) | Supplementary Figure 5-8 |
| Additional analysis | 23 | Give results of additional analyses, if done (such as sensitivity or subgroup analyses, meta-regression [see item 16]) | Figure 3, Supplementary Figure 3-4, 9-10 |
| **Discussion** |  |  |  |
| Summary of evidence | 24 | Summarise the main findings including the strength of evidence for each main outcome; consider their relevance to key groups (such as health care providers, users, and policy makers) | 16-17 |
| Limitations | 25 | Discuss limitations at study and outcome level (such as risk of bias), and at review level (such as incomplete retrieval of identified research, reporting bias) | 18-19 |
| Conclusions | 26 | Provide a general interpretation of the results in the context of other evidence, and implications for future research | 20-21 |
| **Funding** |  |  |  |
| Funding | 27 | Describe sources of funding for the systematic review and other support (such as supply of data) and role of funders for the systematic review | Conflict of interest section, Funding and role of funding source section |

**Source:** Page MJ, McKenzie JE, Bossuyt PM, Boutron I, Hoffmann TC, Mulrow CD, et al. The PRISMA 2020 statement: an updated guideline for reporting systematic reviews. BMJ 2021;372:n71. doi: 10.1136/bmj.n71, For more information, visit: <http://www.prisma-statement.org/>

# **Supplementary Appendix A:** Search strategies

**PubMed** (the most recent search was on Oct 11, 2022)

("albumin bound paclitaxel"[MeSH Terms] OR "albumin bound paclitaxel"[Text Word] OR "paclitaxel albumin bound"[Text Word] OR "protein bound paclitaxel"[Text Word] OR "paclitaxel protein bound"[Text Word] OR "protein bound paclitaxel"[Text Word] OR "Abraxane"[Text Word] OR "ABI007"[Text Word] OR "abi 007"[Text Word] OR "abi 007"[Text Word]) AND ("carcinoma, non small cell lung"[MeSH Terms] OR "carcinoma non small cell lung"[Text Word] OR "carcinomas non small cell lung"[Text Word] OR "lung carcinoma non small cell"[Text Word] OR "lung carcinomas non small cell"[Text Word] OR "Non-Small-Cell Lung Carcinomas"[Text Word] OR "non small cell lung carcinoma"[Text Word] OR "non small cell lung carcinoma"[Text Word] OR "non small cell lung carcinoma"[Text Word] OR "Nonsmall Cell Lung Cancer"[Text Word] OR "carcinoma non small cell lung"[Text Word] OR "Non-Small Cell Lung Cancer"[Text Word] OR “Lung Neoplasms”[MeSH])

**CENTRAL** (the most recent search was on Sep 7, 2022)

"albumin bound paclitaxel"[MeSH Terms] AND ("carcinoma, non small cell lung"[MeSH Terms] OR “Lung Neoplasms”[MeSH])

**Scopus** (the most recent search was on Sep 7, 2022)

( "Albumin-Bound Paclitaxel" OR "Albumin Bound Paclitaxel" OR "Paclitaxel, Albumin-Bound" OR "Protein-Bound Paclitaxel" OR "Paclitaxel, Protein-Bound" OR "Protein Bound Paclitaxel" OR "Abraxane" OR "ABI007" OR "ABI-007" OR "ABI 007" ) AND ( ( "Carcinoma, Non-small cell lung" OR "Carcinoma, Non Small Cell Lung" OR " Carcinomas, Non Small Cell Lung " OR "Lung Carcinoma, Non-Small-Cell" OR "Lung Carcinomas, Non-Small-Cell" OR "Non-Small-Cell Lung Carcinomas" OR "Non-Small Cell Lung Carcinoma" OR " Non-Small-Cell Lung Carcinoma " OR " Non Small Cell Lung Carcinoma " OR " Nonsmall Cell Lung Cancer " OR "Carcinoma, Non-Small Cell Lung " OR " Non-Small Cell Lung Cancer")

**ClinicalTrials.gov** (the most recent search was on Sep 7, 2022)

Condition or disease: NSCLC ("nonsmall cell lung cancer" OR "Non Small Cell Lung Carcinoma" OR "Lung Non-Small Cell Carcinoma" OR "Non-Small Cell Carcinoma of Lung" OR "lung cancer non small cell")

Other terms: nab-paclitaxel ("Albumin Bound Paclitaxel" OR "paclitaxel albumin-stabilized nanoparticle formulation" OR "nanoparticle paclitaxel" OR "Protein Bound Paclitaxel")

**Supplementary Table 2** Characteristics of included comparative studies

| **Study** | **Author** | **Year of publication** | **Sample size Nab/C** | **Study design** | **Study population** | | | **Treatment** | **Control** | **Outcome** | **Duration of follow up (months)** |
| --- | --- | --- | --- | --- | --- | --- | --- | --- | --- | --- | --- |
|  |  |  |  |  | **Cancer**  **stage** | **Gender**  **F/M** | **Age (years)**  **Nab/C** |  |  |  |  |
| 1 | Socinski [1] | 2012 | 521/531 | RCT | IIIB/IV + not received chemotherapy | 263/789 | 60/60 | 100 mg/m^2^ nab-P weekly + C (AUC 6), q 3 weeks (≥6 cycles) | 200 mg/m^2^ sb-P + C (AUC 6) q 3weeks (≥6 cycles) | ORR, CR, PR, SD, PD, PFS, OS | 28 |
| 2 | Liu [2] | 2015 | 55/56 | RCT | IIIB/IV + failed to platinum-based regimen | 40/71 | 52.5/51.1 | 150 mg/m^2^ nab-P q 3 weeks | 500 mg/m^2^ pemetrexed q 3 weeks | CR, PR, SD, PD, PFS, OS | 28 |
| 3 | Wu [3] | 2017 | 46/46 | RCT | III/IV + failed to platinum-based regimen | 42/50 | 58.5/57.2 | 150 mg/m^2^ nab-P q 4 weeks for 2-6 cycles | Matched placebo | ORR, CR, PR, SD, PD, PFS, OS, DCR | 28 |
| 4 | Qin [4] | 2019 | 41/43 | RCT | IIIB/IV + chemotherapy-naïve | 14/70 | 55.4/56 | 100 mg/m^2^ nab-P weekly + 75 mg/m^2^ Cis , q 3 weeks (≥6 cycles) | 1000 mg/m^2^ Gem +  75 mg/m^2^ Cis, q 3 weeks (≥6 cycles) | ORR, CR, PR, SD, PD, PFS, OS, DCR | 50 |
| 5 | Wang [5] | 2019 | 60/64 | RCT | IIIA/IV + chemotherapy-naïve | 11/113 | 58/60 | 135 mg/m^2^ nab-P weekly + C (AUC 5), q 3weeks up to 6 cycles | 1250 mg/m^2^ Gem + C (AUC 5), q 3 weeks up to 6 cycles | ORR, CR, PR, PFS, OS | 96 (median 14.5) |
| 6 | Spigel [6] | 2021 | 136/66 | RCT | IIIB/IV chemotherapy-naïve | 72/130 | 68/68 | 100 mg/m^2^ nab-P weekly + C (AUC 6), q 3weeks x 4 cycles then 100 mg/m^2^ nab-P + best supportive care (BSC) for maintenance | 100 mg/m^2^ nab-P weekly + C (AUC 6), q 3 weeks x 4 cycles then BSC alone for maintenance | PFS, OS, ORR, CR, PR, SD | 57 |
| 7 | Yoneshima [7] | 2021 | 252/251 | RCT | III/IV with previous one or two chemotherapy regiments | 156/347 | 67/68 | 100 mg/m^2^ nab-P q 3 weeks | 60 mg/m^2^ Doc q 3 weeks | OS, PFS, ORR, QoL, Toxicity | 56 (median 15) |
| 8 | Jotte [8] | 2020 | 343/338 | RCT | IV not received chemotherapy for stage IV | 123/558 | 65/66 | 100 mg/m^2^ nab-P weekly + 1,200 mg Atezolizumab+ C (AUC 6), q 3 weeks x 4-6 cycles | 200 mg/m^2^ P weekly + 1,200 mg Atezolizumab + C (AUC 6), q 3 weeks x 4-6 cycles | ORR, DCR, PFS, OS,* Toxicity | 38 |
| 9 | Zhang [9] | 2021 | 46/46 | RCT | IIIA/B + chemotherapy-naïve | 46/46 | 66.5/65.5 | 40mg/m^2^ nab-P weekly + C (AUC 2), q 6 weeks + radiation therapy | 50 mg/m^2^ sb-P + C (AUC 2), q 6 weeks + radiation therapy | OS, ORR, PFS, QoL | 24 |
| 10 | Ai [10] | 2016 | 100/100 | Cohort | untreated stage III/IV NSCLC | 55/145 | 57.8/58.5 | 260 mg/m^2^ nab-P weekly + 75 mg/m^2^ Cis, q 3 weeks at least 2 cycles | 1,000 mg/m^2^ Gem weekly + 75 mg/m^2^ Cis, q 3 weeks at least 2 cycles | CR, PR, SD, PD, RR, DCR, PFS | 50 |
| 11 | Chen [11] | 2017 | 55/216 | Cohort | untreated stage IIIB/IV NSCLC | 465/225 | 59/57 | 130 mg/m^2^ nab-P weekly + Cis, Q3weeks | 75 mg/m^2^ Doc + Cis, Q3weeks | CR, PR, SD, PD, OS, RR, ORR, DCR, PFS | 90 |

**Note:** *Three-arm study in which all efficacy outcomes were reported in arms not relevant to our study.

**Abbreviations**: AUC, area under the curve; C, carboplatin; Cis, cisplatin; Doc, docetaxel; G, gemcitabine; RCTs, nab-P, albumin-bound paxlitaxel nanoparticle; randomized controlled trials; sb-P, solvent-based paclitaxel; CR, complete remission; PR, partial response; SD, stable disease; PD, progressive disease; RR, response rate; OR, overall survival; ORR, objective response rate; DCR, disease control rate; PFS, progression-free survival; QoL, quality of life; q, every

**Supplementary Table 3** Characteristics of included non-comparative studies

| Study characteristics | | | | | | | | Study population | | Outcome |
| --- | --- | --- | --- | --- | --- | --- | --- | --- | --- | --- |
| Study | Author | Year of publication | Research type | Sample size | Study design | Dosage and frequency | Study phase/stage | Gender  F/M | Average age |  |
| 1 | Green [12] | 2006 | Trial | 43 | phase II multicenter | 260 mg/m^2^ Q3weeks | IIIB/IV | 10/33 | 58 | OR, RR, CR, PR, SD, PD, death |
| 2 | Rizvi [13] | 2008 | Trial | 40 | phase II | 125 mg/m^2^ Q4weeks | IV | 21/19 | 70 | OS, RR, CR, PR, PFS |
| 3 | Zheng [14] | 2012 | Cohort | 20 | NR | 100 mg/m^2^ Q4weeks | IV | 9/11 | 66 | CR, PR, SD, OR, PD, ORR, DCR |
| 4 | Yuan [15] | 2012 | Cohort | 33 | phase I/II | 260 mg/m^2^ Q3weeks | IV | 14/19 | 54 | CR, PR, SD, OR, PD, PFS, DCR, ORR |
| 5 | Hu [16] | 2015 | Trial | 56 | phase II | 100 mg/m^2^ Q4weeks | IIIB/IV | 25/31 | 59.6 | CR, PR, SD, ORR, PD, PFS, DCR |
| 6 | Lin [17] | 2016 | Cohort (Chinese) | 69 | NR | 130 mg/m^2^ Q3weeks | IIIB/IV | 27/42 | 64.8 | CR, PR, SD, PD, ORR, DCR, PFS |
| 7 | Jin[18] | 2016 | Cohort | 42 | NR | 100 mg/m^2^ Q4weeks | III/IV | 5/37 | 71 | CR, PR, SD, OS, PFS, DCR, ORR |
| 8 | Sakata [19] | 2016 | Trial | 41 | phase II | 100 mg/m^2^ Q3weeks | IIIB/IV | 18/23 | 68 | CR, PR, SD, OS, PFS, DCR, ORR, PFS |
| 9 | Duan [20] | 2017 | Cohort | 62 | NR | 130 mg/m^2^ Q3weeks | IV | 27/35 | NR | CR, PR, SD, PD, ORR, DCR, PFS, OS |
| 10 | Li [21] | 2017 | Cohort (Chinese) | 50 | NR | 130 mg/m^2^ Q3weeks | III/IV | 11/39 | NR | CR, PR, SD, PD, ORR, DCR, PFS |
| 11 | Wong [22] | 2017 | Cohort | 34 | NR | 260 mg/m^2^ Q3weeks | III/IV | 8/26 | NR | CR, PR, SD, PD, ORR, DCR, PFS, OS |
| 12 | Tanaka [23] | 2017 | Trial | 31 | Phase II | 100 mg/m^2^ Q4weeks | IIIB/IV | 7/24 | 66 | CR, PR, SD, OS, ORR, DCR, PFS |
| 13 | Xing [24] | 2017 | Trial | 98 | NR | 130 mg/m^2^ Q3weeks | IIIB/IV | 28/70 | 61 | CR, PR, SD, PD, OS, ORR, DCR, PFS |
| 14 | Anzai [25] | 2017 | Trial | 32 | Phase II | 100 mg/m^2^ Q4weeks | IIIB/IV | 8/24 | 67.5 | CR, PR, SD, PD, RR, DCR, PFS, OS |
| 15 | Xiao [26] | 2018 | Cohort (Chinese) | 56 | NR | 260 mg/m^2^ Q3weeks | NR | NR | NR | CR, PR, SD, PD, RR, DCR, PFS |
| 16 | Harada [27] | 2019 | Trial | 55 | Phase II | 100 mg/m^2^ Q4weeks | IIB/IV | 15/40 | 66 | CR, PR, SD, DC, OS, ORR, DCR, PFS |
| 17 | Kato [28] | 2019 | Trial | 22 | Phase II | 100 mg/m^2^ Q3weeks | IIB/IV | 6/16 | 65 | CR, PR, SD, PD, DCR, OS, ORR, PFS |
| 18 | Kotake [29] | 2020 | Trial | 35 | Phase II | 100 mg/m^2^ Q4weeks | IIIA-IV | 10/25 | 69 | CR, PR, SD, PD, DCR, OS, ORR, PFS |
| 19 | Weiss [30] | 2022 | Trial | 42 | Phase II | 100 mg/m^2^ Q4weeks | IV | 22/20 | 76.3 | OR, CR, PR, SD, DC, OS, PFS |
| 20 | Wang [31] | 2020 | Cohort | 76 | NR | 150 mg/m^2^ Q3weeks | IIB/IV | 24/52 | 72 | ORR, CR, PR, SD, DCR, OS, PFS |
| 21 | Baik [32] | 2021 | Trial | 27 | Phase II | 125 mg/m^2^ Q4weeks | IIIB/IV | 18/8 | 65 | ORR, PR, SD, DC PD, OS, PFS |
| 22 | Miyauchi [33] | 2021 | Trial | 18 | Phase II | 150 mg/m^2^ Q3weeks | IV or postoperative recurrent NSCLC | 3/15 | 68 | ORR, PR, CR, PD, OS, PFS |
| 23 | Yoshimura [34] | 2021 | Trial | 30 | Phase II | 80 mg/m^2^ Q3weeks | IIIB/IV | 7/23 | 65 | ORR, PR, SD, DC PD, OR, OS, DCR, PFS |
| 24 | Shoji [35] | 2022 | Trial | 65 | Phase II | 100 mg/m^2^ Q4weeks | IIIB/IV | 15/50 | 69 | ORR, OS, PFS, DCR |

**Abbreviations:** AUC, area under the curve; C, carboplatin; Cis, cisplatin; Doc, docetaxel; G, gemcitabine; RCTs, nab-P, albumin-bound paxlitaxel nanoparticle; randomized controlled trials; sb-P, solvent-based paclitaxel; CR, complete remission; NR, not report; NSCLC, non-small cell lung cancer; PR, partial response; SD, stable disease; PD, progressive disease; RR, response rate; OR, overall survival; ORR, objective response rate; DCR, disease control rate; PFS, progression-free survival; QoL, quality of life; q, every

**Supplementary Figure 1** Risk of bias of individual RCT


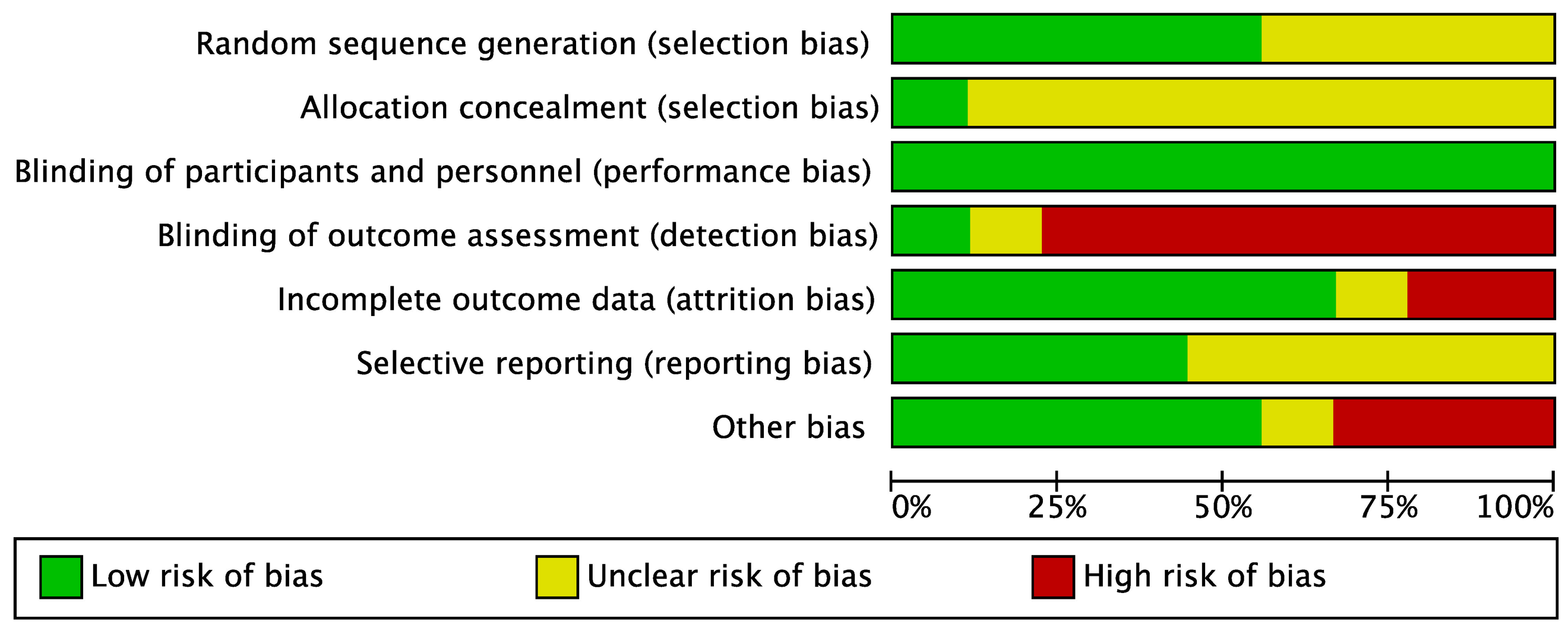


**Supplementary Figure 2** Summary risk of bias of included RCTs

# **Supplementary Appendix B:** NOS key criteria for assessment

**Newcastle - Ottawa quality assessment scale (NOS) for comparative cohort studies**

**Key criteria for assessment**

**Selection (4 out of 4)**

*1) Representativeness of the exposed cohort*

a) Truly representative of NSCLC patients*

b) Somewhat representative of NSCLC patients *

c) Selected group of users e.g., nurses, volunteers, or only certain socioeconomic groups/ areas

d) No description of the derivation of the cohort

*2) Selection of the non-exposed cohort*

a) Drawn from the same hospital as the exposed cohort *

b) Drawn from a different source

c) No description of the derivation of the non-exposed cohort

*3) Ascertainment of exposure (i.e., chemotherapy regimen)*

a) Secure record (e.g., surgical records, health care records, or used standard laboratory procedure) *

b) Structured interview *

c) Written self-report

d) No description

*4) Demonstration that outcome of interest (i.e., survival and response) was not present at start of study and outcome definition was clearly defined.*

a) Yes *

b) No

**Comparability (2 out of 2)**

*1) Comparability of cohorts on the basis of the design or analysis*

a) Characteristics are comparable between groups and/or study controls for a tumor, node, metastasis (TNM) stage of cancer at presentation *

b) Study controls for any additional factors in addition to a)*

**Outcome (3 out of 3)**

*1) Assessment of outcome (i.e., survival and response)*

a) Independent blind assessment or confirmation of the outcome by reference to secure records *

b) Record linkage (e.g., identified through ICD codes on database records) *

c) Self-report (i.e., no reference to original health records or documented source to confirm the outcome)

d) No description

*2) Was follow-up long enough for outcomes to occur*

a) Yes (if mean or median follow-up period ≥ 48 months) *

b) No (if mean or median follow-up period < 48 months)

*3) Adequacy of follow up of cohorts*

a) Complete follow up – all subjects accounted for *

b) Subjects LTFU unlikely to introduce bias small number lost (i.e., ≥ 80%) or description provided of those lost *

c) Follow up rate < 80% and no description of those lost

d) No statement

**Supplementary Table 4** Quality assessment of included comparative cohort studies

| **Author, Year** | **Selection (4)** | | | | **Compar-ability (2)** | | **Outcome (3)** | | | **Total Score (9)** |
| --- | --- | --- | --- | --- | --- | --- | --- | --- | --- | --- |
|  | **Representativeness** | **Selection of non-exposed cohort** | **Ascertainment of exposure** | **Outcome of interest not present at baseline** | **Controlled for main factors** | **Controlled for additional factors** | **Assessment of outcome** | **Length of follow-up** | **Adequacy of follow-up** |  |
| 1. Ai, 2016 | 1 | 1 | 1 | 1 | 1 | 0 | 1 | 1 | 1 | 8 |
| 2. Chen, 2017 | 0 | 1 | 1 | 1 | 1 | 0 | 1 | 1 | 0 | 6 |

**Note:** A study with total score of ≥7 with no individual component score of 0 will be considered as “good quality”.

**Supplementary Table 5** Risk of bias of included non-comparative trials

| Study,  publication year | A clearly stated aim | Inclusion of consecutive patients | Prospective collection of data | Endpoints appropriate to the aim of the study | Unbiased assessment of the study endpoint | Follow-up period appropriate to the aim of the study | Loss to follow-up less than 5% | Prospective calculation of the study size | Total |
| --- | --- | --- | --- | --- | --- | --- | --- | --- | --- |
| 1. Green, 2006 | 2 | 2 | 2 | 2 | 0 | 2 | 0 | 0 | 10 |
| 2. Rizvi, 2008 | 2 | 2 | 2 | 1 | 2 | 2 | 2 | 2 | 15 |
| 3. Hu, 2015 | 2 | 2 | 2 | 1 | 0 | 2 | 0 | 2 | 11 |
| 4. Sakata, 2016 | 2 | 2 | 2 | 1 | 2 | 2 | 0 | 2 | 13 |
| 5. Tanaka, 2017 | 2 | 2 | 2 | 2 | 2 | 2 | 0 | 2 | 14 |
| 6. Xing, 2017 | 2 | 2 | 2 | 1 | 0 | 2 | 0 | 0 | 9 |
| 7. Anzai, 2017 | 2 | 2 | 2 | 2 | 0 | 2 | 0 | 2 | 12 |
| 8. Harada, 2019 | 2 | 2 | 2 | 1 | 2 | 2 | 0 | 2 | 13 |
| 9. Kato, 2019 | 2 | 2 | 2 | 1 | 2 | 2 | 0 | 2 | 13 |
| 10. Kotake, 2020 | 2 | 2 | 2 | 1 | 0 | 2 | 0 | 2 | 11 |
| 11. Weiss, 2022 | 2 | 2 | 2 | 1 | 0 | 2 | 0 | 2 | 11 |
| 12. Baik, 2021 | 2 | 2 | 2 | 2 | 0 | 2 | 2 | 2 | 14 |
| 13. Miyauchi, 2021 | 2 | 2 | 2 | 1 | 2 | 2 | 0 | 2 | 13 |
| 14. Yoshimurs, 2021 | 2 | 2 | 2 | 2 | 0 | 2 | 0 | 2 | 12 |
| 15. Shoji, 2022 | 2 | 2 | 2 | 1 | 2 | 2 | 0 | 2 | 13 |

| A) | ** |
| --- | --- |
| B) | ** |
| C) | ** |

**Supplementary Figure 3** Subgroup analysis of progress-free survival (PFS) according to included RCTs

Note: Subgrouped by A) comparison, B) treatment order, and C) histology

| A) | ** | B) | ** |
| --- | --- | --- | --- |
| C) | ** |  |  |

**Supplementary Figure 4** Subgroup analysis of overall survival (OS) according to included RCTs

Note: Subgrouped by A) comparison, B) treatment order, and C) histology

**Supplementary Table 6** Cumulative incidence of efficacy outcomes according to non-comparative studies

| **Efficacy** | **Design** | **Studies** | **Events/ Total** | **Model** | **Cumulative incidence (95% CI)** | **I^2^, P-value** |
| --- | --- | --- | --- | --- | --- | --- |
| Overall response | Trial | 15 | 143/ 634 | Fixed | 0.22 (0.19, 0.25) | 37.6%, 0.070 |
|  |  |  |  | Random | 0.22 (0.18, 0.27) |  |
|  | Cohort | 9 | 98/ 442 | Fixed | 0.22 (0.18, 0.26) | 23.8%, 0.23 |
|  |  |  |  | Random | 0.22 (0.18, 0.27) |  |
| Disease control | Trial | 15 | 405/ 640 | Fixed | 0.64 (0.60, 0.67) | 64.4%, <0.001 |
|  |  |  |  | Random | 0.64 (0.58, 0.70) |  |
|  | Cohort | 9 | 285/ 442 | Fixed | 0.65 (0.60, 0.69) | 60.6%, 0.009 |
|  |  |  |  | Random | 0.64 (0.57, 0.71) |  |
| Death | Trial | 11 | 29/ 474 | Fixed | 0.02 (0.01, 0.03) | 90.1%, <0.001 |
|  |  |  |  | Random | 0.02 (0.00, 0.08) |  |
|  | Cohort | 4 | 0/ 200 | Fixed | Not converged | Not available |
|  |  |  |  | Random | Not converged |  |

**Abbreviations:** CI; confidence interval, NA; not available

**Supplementary Table 7** Cumulative incidence of safety outcomes according to non-comparative studies

| **Safety profile** | **Study design** | **Studies** | **Events/ Total** | **Model** | **Cumulative incidence (95% CI)** | **I^2^, P-value** |
| --- | --- | --- | --- | --- | --- | --- |
| **Hematologic** |  |  |  |  |  |  |
| Neutropenia | Trial | 14 | 159/ 581 | Fixed | 0.26 (0.22, 0.29) | 83.2%, <0.001 |
|  |  |  |  | Random | 0.25 (0.16, 0.34) |  |
|  | Cohort | 9 | 36/ 391 | Fixed | 0.07 (0.04, 0.10) | 82.2%, <0.001 |
|  |  |  |  | Random | 0.06 (0.02, 0.13) |  |
| Leukopenia | Trial | 15 | 79/ 634 | Fixed | 0.08 (0.06, 0.10) | 90.1%, <0.001 |
|  |  |  |  | Random | 0.10 (0.04, 0.19) |  |
|  | Cohort | 7 | 24/ 264 | Fixed | 0.08 (0.05, 0.12) | 29.2%, 0.20 |
|  |  |  |  | Random | 0.09 (0.05, 0.13) |  |
| Thrombocytopenia | Trial | 15 | 6/ 654 | Fixed | 0.00 (0.00, 0.01) | 37.5%, 0.07 |
|  |  |  |  | Random | 0.00 (0.00, 0.01) |  |
|  | Cohort | 8 | 3/ 322 | Fixed | 0.00 (0.00, 0.01) | 12.8%, 0.33 |
|  |  |  |  | Random | 0.00 (0.00, 0.01) |  |
| Anemia | Trial | 15 | 27/ 654 | Fixed | 0.02 (0.01, 0.04) | 70.3%, <0.001 |
|  |  |  |  | Random | 0.03 (0.01, 0.06) |  |
|  | Cohort | 6 | 2/ 243 | Fixed | 0.00 (0.00, 0.01) | 0.0%, 0.42 |
|  |  |  |  | Random | 0.00 (0.00, 0.01) |  |
| **Non hematologic** |  |  |  |  |  |  |
| Sensory neuropathy | Trial | 13 | 36/ 589 | Fixed | 0.04 (0.03, 0.06) | 78.2%, <0.001 |
|  |  |  |  | Random | 0.05 (0.02, 0.09) |  |
|  | Cohort | 6 | 9/ 243 | Fixed | 0.02 (0.01, 0.04) | 67.1%, 0.010 |
|  |  |  |  | Random | 0.03 (0.00, 0.07) |  |
| Arthralgia | Trial | 11 | 6/ 465 | Fixed | 0.00 (0.00, 0.01) | 41.8%, 0.070 |
|  |  |  |  | Random | 0.00 (0.00, 0.01) |  |
|  | Cohort | 7 | 0/ 319 | Fixed | Not converged | Not available |
|  |  |  |  | Random | Not converged |  |

**Abbreviations:** CI; confidence interval, NA; not available

| (A)   | (B)   | (C)   |
| --- | --- | --- |
| Original pooled RR = 1.35 (1.19, 1.53)  Trim-and-fill pooled RR = 1.33 (1.17, 1.51) | Original pooled RR = 1.11 (0.98, 1.26)  Trim-and-fill pooled RR = 1.06 (0.90, 1.25) | Original pooled RR = 1.64 (0.40, 6.67)  Trim-and-fill pooled RR = no imputed study |
| (D)   | (E)   | (F)   |
| Original pooled RR = 1.34 (1.18, 1.53)  Trim-and-fill pooled RR = 1.33 (1.17, 1.51) | Original pooled RR = 0.94 (0.83, 1.06)  Trim-and-fill pooled RR = 0.92 (0.81, 1.03) | Original pooled RR = 0.76 (0.64, 0.90)  Trim-and-fill pooled RR = no imputed study |
|  |  |  |
| (G)   | (H)   |  |
| Original pooled HR = 0.90 (0.81, 0.99)  Trim-and-fill pooled HR = no imputed study | Original pooled HR = 0.84 (0.76, 0.93)  Trim-and-fill pooled HR = 0.87 (0.79, 0.95) |  |

**Supplementary Figure 5** Contour-enhanced funnel plots of efficacy outcomes according to included RCTs

| (A)   | (B)   | (C)   |
| --- | --- | --- |
| Original pooled cumulative incidence = 0.22 (0.18, 0.27)  Trim-and-fill pooled CI = 0.17 (0.12, 0.21) | Original pooled cumulative incidence = 0.22 (0.18, 0.27)  Trim-and-fill pooled CI = 0.19 (0.14, 0.24) | Original pooled cumulative incidence = 0.64 (0.60, 0.67)  Trim-and-fill pooled CI = no imputed study |
| (D)   | (E)   |  |
| Original pooled cumulative incidence = 0.64 (0.57, 0.71)  Trim-and-fill pooled CI = no imputed study | Original pooled cumulative incidence = 0.02 (0.00, 0.08)  Trim-and-fill pooled CI = no imputed study |  |

**Supplementary Figure 6** Funnel plots of efficacy outcomes according to non-comparative studies

| (A)   | (B)   | (C)   |
| --- | --- | --- |
| Original pooled RR = 1.19 (0.66, 2.16)  Trim-and-fill pooled RR = 0.95 (0.55, 1.64) | Original pooled RR = 1.33 (0.47, 3.75)  Trim-and-fill pooled RR = not converged | Original pooled RR = 1.23 (0.75, 2.03)  Trim-and-fill pooled RR = no imputed study |
| (D)   | (E)   |  |
| Original pooled RR = 1.42 (0.83, 2.41)  Trim-and-fill pooled RR = 1.51 (0.90, 2.56) | Original pooled RR = 3.36 (0.57, 19.95)  Trim-and-fill pooled RR = 2.38 (0.47, 11.98) |  |

**Supplementary Figure 7** Contour-enhanced funnel plots of safety outcomes according to included RCTs

| (A)   | (B)   | (C)   |
| --- | --- | --- |
| Original pooled cumulative incidence = 0.25 (0.16, 0.34)  Trim-and-fill pooled CI = no imputed study | Original pooled cumulative incidence = 0.06 (0.02, 0.13)  Trim-and-fill pooled CI = 0.03 (-0.01, 0.08) | Original pooled cumulative incidence = 0.10 (0.04, 0.19)  Trim-and-fill pooled CI = 0.04 (0.003, 0.08) |
| (D)   | (E)   | (F)   |
| Original pooled cumulative incidence = 0.08 (0.05, 0.12)  Trim-and-fill pooled CI = 0.06 (0.03, 0.09) | Original pooled cumulative incidence = 0.00 (0.00, 0.01)  Trim-and-fill pooled CI = no imputed study | Original pooled cumulative incidence = 0.00 (0.00, 0.01)  Trim-and-fill pooled CI = 0.003 (-0.009, 0.015) |

| (G)   | (H)   | (I)   |
| --- | --- | --- |
| Original pooled cumulative incidence = 0.03 (0.01, 0.06)  Trim-and-fill pooled CI = 0.01 (-0.01, 0.03) | Original pooled cumulative incidence = 0.00 (0.00, 0.01)  Trim-and-fill pooled CI = no imputed study | Original pooled cumulative incidence = 0.05 (0.02, 0.09)  Trim-and-fill pooled CI = 0.02 (-0.01, 0.04) |
| (J)   | (K)   |  |
| Original pooled cumulative incidence = 0.03 (0.00, 0.07)  Trim-and-fill pooled CI = 0.004 (-0.01, 0.02) | Original pooled cumulative incidence = 0.00 (0.00, 0.01)  Trim-and-fill pooled CI = no imputed study |  |

**Supplementary Figure 8** Funnel plots of safety outcomes according to non-comparative studies

| A) |  | B) |  |
| --- | --- | --- | --- |
| C) |  | D) |  |

**Supplementary Figure 9** Leave-one-out analysis of efficacy outcomes according to comparative studies

Note: A) overall response, B) partial response, C) progressive disease, and D) progress-free survival

|  |  |
| --- | --- |
|  |  |
|  |  |

**Supplementary Figure 10** Leave-one-out analysis of safety outcomes according to comparative studies

# **Supplementary Appendix C**: eReferences

[1] M.A. Socinski, I. Bondarenko, N.A. Karaseva, A.M. Makhson, I. Vynnychenko, I. Okamoto, J.K. Hon, V. Hirsh, P. Bhar, H. Zhang, J.L. Iglesias, M.F. Renschler, Weekly nab-paclitaxel in combination with carboplatin versus solvent-based paclitaxel plus carboplatin as first-line therapy in patients with advanced non-small-cell lung cancer: final results of a phase III trial, J Clin Oncol. 30 (2012) 2055–2062. https://doi.org/10.1200/JCO.2011.39.5848.

[2] Z. Liu, Z. Wei, Y. Hu, F. Gao, L. Hao, P. Fang, S. Sun, J. Li, S. Jiao, A phase II open-label clinical study of comparing nab-paclitaxel with pemetrexed as second-line chemotherapy for patients with stage IIIB/IV non-small-cell lung cancer, Med Oncol. 32 (2015). https://doi.org/10.1007/s12032-015-0660-5.

[3] Y. Wu, J. Feng, W. Hu, Q. Luo, A randomized placebo-controlled clinical study of nab-paclitaxel as second-line chemotherapy for patients with advanced non-small cell lung cancer in China, Biosci Rep. 37 (2017) 1–6. https://doi.org/10.1042/BSR20170020.

[4] S. Qin, H. Yu, X. Wu, Z. Luo, H. Wang, S. Sun, M. Huang, J. Jin, Z. Tao, J. Qiao, Y. Feng, J. Wang, J. Chang, Weekly albumin-bound paclitaxel/cisplatin versus gemcitabine/cisplatin as first-line therapy for patients with advanced non-small-cell lung cancer: a phase II open-label clinical study, Chin J Cancer Res. 31 (2019) 339–348. https://doi.org/10.21147/j.issn.1000-9604.2019.02.08.

[5] Z. Wang, C. Huang, J.J. Yang, Y. Song, Y. Cheng, G.Y. Chen, H.H. Yan, X.S. Ben, B.C. Wang, C.R. Xu, B.Y. Jiang, Q. Zhou, H.J. Chen, Y.L. Wu, A randomised phase II clinical trial of nab-paclitaxel and carboplatin compared with gemcitabine and carboplatin as first-line therapy in advanced squamous cell lung carcinoma (C-TONG1002), Eur J Cancer. 109 (2019) 183–191. https://doi.org/10.1016/j.ejca.2019.01.007.

[6] D.R. Spigel, R.M. Jotte, S.P. Aix, L. Gressot, D. Morgensztern, M. McCleod, M.A. Socinski, D. Daniel, O. Juan-Vidal, K.F. Mileham, H. West, R. Page, N. Reinmuth, J. Knoble, T. Chen, R. Bhore, M. Wolfsteiner, T.J. Ong, C. Gridelli, M. Thomas, Nanoparticle albumin-bound paclitaxel plus carboplatin induction followed by nanoparticle albumin-bound paclitaxel maintenance in squamous non–small-cell lung cancer (ABOUND.SQM): a phase III randomized clinical trial, Clin Lung Cancer. 22 (2021) 6-15.e4. https://doi.org/10.1016/j.cllc.2020.09.007.

[7] Y. Yoneshima, S. Morita, M. Ando, A. Nakamura, S. Iwasawa, H. Yoshioka, Y. Goto, M. Takeshita, T. Harada, K. Hirano, T. Oguri, M. Kondo, S. Miura, Y. Hosomi, T. Kato, T. Kubo, J. Kishimoto, N. Yamamoto, Y. Nakanishi, I. Okamoto, Phase 3 Trial Comparing Nanoparticle Albumin-Bound Paclitaxel With Docetaxel for Previously Treated Advanced NSCLC, Journal of Thoracic Oncology. 16 (2021) 1523–1532. https://doi.org/10.1016/j.jtho.2021.03.027.

[8] R. Jotte, F. Cappuzzo, I. Vynnychenko, D. Stroyakovskiy, D. Rodríguez-Abreu, M. Hussein, R. Soo, H.J. Conter, T. Kozuki, K.-C. Huang, V. Graupner, S.W. Sun, T. Hoang, H. Jessop, M. McCleland, M. Ballinger, A. Sandler, M.A. Socinski, Atezolizumab in combination with carboplatin and nab-paclitaxel in advanced squamous NSCLC (IMpower131): results from a randomized phase III trial, J Thorac Oncol. 15 (2020) 1351–1360. https://doi.org/10.1016/j.jtho.2020.03.028.

[9] Y. Zhang, Phase I/II nab paclitaxel, paclitaxel & carboplatin with RTX followed by consolidation in patients with favorable prognosis inoperable stage IIIA/B NSCLC, ClinicalTrials.Gov. (2021). https://clinicaltrials.gov/ct2/show/results/NCT01757288 (accessed March 18, 2023).

[10] D. Ai, Y. Guan, X.J. Liu, C.F. Zhang, P. Wang, H.L. Liang, Q. Sen Guo, Clinical comparative investigation of efficacy and toxicity of cisplatin plus gemcitabine or plus abraxane as first-line chemotherapy for stage III/IV non-small-cell lung cancer, Onco Targets Ther. 9 (2016) 5693–5698. https://doi.org/10.2147/OTT.S109683.

[11] Y. Chen, J. Li, S. Chen, Y. Zhang, Y. Hu, G. Zhang, X. Yan, S. Jiao, Nab-Paclitaxel in combination with Cisplatin Versus Docetaxel Plus Cisplatin as First-Line Therapy in Non-small Cell Lung Cancer, Sci Rep. 7 (2017) 1–7. https://doi.org/10.1038/s41598-017-11404-9.

[12] M.R. Green, G.M. Manikhas, S. Orlov, B. Afanasyev, A.M. Makhson, P. Bhar, M.J. Hawkins, Abraxane®, a novel Cremophor®-free, albumin-bound particle form of paclitaxel for the treatment of advanced non-small-cell lung cancer, Annals of Oncology. 17 (2006) 1263–1268. https://doi.org/10.1093/annonc/mdl104.

[13] N.A. Rizvi, G.J. Riely, C.G. Azzoli, V.A. Miller, K.K. Ng, J. Fiore, G. Chia, M. Brower, R. Heelan, M.J. Hawkins, M.G. Kris, Phase I/II trial of weekly intravenous 130-nm albumin-bound paclitaxel as initial chemotherapy in patients with stage IV non-small-cell lung cancer, Journal of Clinical Oncology. 26 (2008) 639–643. https://doi.org/10.1200/JCO.2007.10.8605.

[14] Q. Zheng, Y. Yao, K. Nan, Weekly intravenous nanoparticle albumin-bound paclitaxel for elderly patients with stage IV non-small-cell lung cancer: A series of 20 cases, J Biomed Res. 26 (2012) 159–164. https://doi.org/10.7555/JBR.26.20110106.

[15] D. mei Yuan, Y. ling Lv, Y. wen Yao, X. hui Miao, Q. Wang, X. wu Xiao, J. Yin, Y. Shi, M. qi Shi, X. wei Zhang, Y. Song, Efficacy and safety of Abraxane in treatment of progressive and recurrent non-small cell lung cancer patients: A retrospective clinical study, Thorac Cancer. 3 (2012) 341–347. https://doi.org/10.1111/j.1759-7714.2012.00113.x.

[16] W. Hu, Z. Zhang, A phase II clinical study of using nab-paclitaxel as second-line chemotherapy for Chinese patients with advanced non-small cell lung cancer, Medical Oncology. 32 (2015) 1–5. https://doi.org/10.1007/s12032-015-0498-x.

[17] L. Lin, Z. Ping, W. Hui, Z. Zijin, W. Xiaonan, Albumin bound paclitaxel for the treatment of retreatment advanced non-small-cell lung cancer, Cancer Research and Clinic. 28 (2016). https://doi.org/10.3760/cma.j.issn.1006-9801.2016.05.0l0.

[18] F. Jin, H. Zhu, F. Shi, L. Kong, J. Yu, A retrospective analysis of safety and efficacy of weekly nab-paclitaxel as second-line chemotherapy in elderly patients with advanced squamous non-small-cell lung carcinoma, Clin Interv Aging. 11 (2016) 167–173. https://doi.org/10.2147/CIA.S97363.

[19] S. Sakata, S. Saeki, I. Okamoto, K. Otsubo, K. Komiya, R. Morinaga, Y. Yoneshima, Y. Koga, A. Enokizu, H. Kishi, S. Hirosako, E. Yamaguchi, N. Aragane, S. Fujii, T. Harada, E. Iwama, H. Semba, Y. Nakanishi, H. Kohrogi, Phase II trial of weekly nab-paclitaxel for previously treated advanced non–small cell lung cancer: Kumamoto thoracic oncology study group (KTOSG) trial 1301, Lung Cancer. 99 (2016) 41–45. https://doi.org/10.1016/j.lungcan.2016.06.009.

[20] J. Duan, Y. Hao, R. Wan, S. Yu, H. Bai, T. An, J. Zhao, Z. Wang, M. Zhuo, J. Wang, Efficacy and safety of weekly intravenous nanoparticle albumin-bound paclitaxel for non-small cell lung cancer patients who have failed at least two prior systemic treatments, Thorac Cancer. 8 (2017) 138–146. https://doi.org/10.1111/1759-7714.12413.

[21] X. Li, B. Ai, P. Zhang, L. Li, X. Wu, Clinical research on albumin-bound paclitaxel-based therapy in advanced lung cancer, Chinese Journal of Lung Cancer. 20 (2017) 479–484. https://doi.org/10.3779/j.issn.1009-3419.2017.07.07.

[22] W. Wong, P. Sun, Z. Mu, J. Liu, C. Yu, A. Liu, Efficacy and safety of nab-paclitaxel as second-line chemotherapy for locally advanced and metastatic non-small cell lung cancer, in: Anticancer Res, 2017. https://doi.org/10.21873/anticanres.11873.

[23] H. Tanaka, K. Taima, T. Morimoto, Y. Tanaka, M. Itoga, K. Nakamura, A. Hayashi, M. Kumagai, H. Yasugahira, M. Mikuniya, K. Okudera, S. Takanashi, S. Tasaka, A single-arm phase II study of nab-paclitaxel for patients with chemorefractory non-small cell lung cancer, BMC Cancer. 17 (2017) 1–6. https://doi.org/10.1186/s12885-017-3684-8.

[24] P. Xing, Y. Zhu, L. Shan, S. Chen, X. Hao, J. Li, The role of weekly nanoparticle albumin bound paclitaxel monotherapy as second line or later treatment for advanced NSCLC in China, Oncotarget. 8 (2017) 87442–87454. https://doi.org/10.18632/oncotarget.21103.

[25] M. Anzai, M. Morikawa, T. Okuno, Y. Umeda, Y. Demura, T. Sonoda, M. Yamaguchi, K. Kanno, K. Shiozaki, S. Ameshima, M. Akai, T. Ishizuka, Efficacy and safety of nanoparticle albumin-bound paclitaxel monotherapy as second-line therapy of cytotoxic anticancer drugs in patients with advanced non-small cell lung cancer, Medicine (United States). 96 (2017). https://doi.org/10.1097/MD.0000000000009320.

[26] J. Xiao, W. Dong, J. Peng, X. Jiang, Z.F. Yin, H. Li, Clinical observation of albumin-binding paclitaxel monotherapy in the treatment of elderly patients with advanced non-small cell lung cancer, Cancer Research and Clinic. 30 (2018). https://doi.org/10.3760/cma.j.issn.1006-9801.2018.01.012.

[27] D. Harada, T. Kozuki, N. Nogami, A. Bessho, S. Hosokawa, N. Fukamatsu, K. Hotta, K. Ohashi, T. Kubo, H. Yoshioka, T. Yokoyama, N. Sone, S. Kuyama, K. Kudo, M. Yasugi, N. Takigawa, I. Oze, K. Kiura, A phase I/II trial of weekly nab-paclitaxel for pretreated non-small-cell lung cancer patients without epidermal growth factor receptor mutations and anaplastic lymphoma kinase rearrangement, Asia Pac J Clin Oncol. 15 (2019) 250–256. https://doi.org/10.1111/ajco.13147.

[28] Y. Kato, Y. Okuma, K. Watanabe, M. Yomota, S. Kawai, Y. Hosomi, T. Okamura, A single-arm phase II trial of weekly nanoparticle albumin-bound paclitaxel (nab-paclitaxel) monotherapy after standard of chemotherapy for previously treated advanced non-small cell lung cancer, Cancer Chemother Pharmacol. 84 (2019) 351–358. https://doi.org/10.1007/s00280-019-03843-0.

[29] M. Kotake, T. Kuwako, H. Imai, Y. Tomizawa, K. Kaira, A. Yoshii, M. Ochiai, Y. Miura, T. Osaki, R. Sakurai, K. Takei, K. Minato, R. Saito, Phase II Study of Weekly Nanoparticle Albumin-Bound Paclitaxel as Second- or Third-Line Therapy in Patients with Advanced Non-Small Cell Lung Cancer, Chemotherapy. 65 (2020) 21–28. https://doi.org/10.1159/000508715.

[30] J.M. Weiss, N. Pennell, A.M. Deal, D. Morgensztern, D.S. Bradford, J. Crane, H.J. West, C. Lee, C. Pecot, J.P. Stevenson, W. Irvin, M. Socinski, T. Stinchcombe, L.C. Villaruz, H.B. Muss, Nab-paclitaxel in older patients with non–small cell lung cancer who have developed disease progression after platinum-based doublet chemotherapy, Cancer. 126 (2020) 1060–1067. https://doi.org/10.1002/cncr.32573.

[31] S. Wang, Q. Liang, Y. Chi, M. Zhuo, T. An, J. Duan, Z. Wang, Y. Wang, J. Zhong, X. Yang, H. Chen, J. Wang, J. Zhao, Retrospective analysis of the effectiveness and tolerability of nab-paclitaxel in Chinese elderly patients with advanced non-small-cell lung carcinoma, Thorac Cancer. 11 (2020) 1149–1159. https://doi.org/10.1111/1759-7714.13356.

[32] C. Baik, S. Lee, K. Cook, S. Wallace, R. Wood, R. Santana-Davila, L. Chow, C. Rodriguez, K.D. Eaton, R. Martins, A Phase II study of nab-Paclitaxel (nab-P) in patients with advanced non-small cell lung cancer with EGFR mutations after frontline tyrosine kinase inhibitor therapy, Cancer Treat Res Commun. 28 (2021) 100416. https://doi.org/10.1016/j.ctarc.2021.100416.

[33] E. Miyauchi, H. Tanaka, A. Nakamura, T. Harada, T. Nakagawa, M. Morita, D. Jingu, T. Kuda, S. Gamou, R. Saito, A. Inoue, Phase I/II study of biweekly nab-paclitaxel in patients with platinum-pretreated non-small cell lung cancer: NJLCG1402, Thorac Cancer. 12 (2021) 2886–2893. https://doi.org/10.1111/1759-7714.14149.

[34] N. Yoshimura, K. Sawa, T. Nakai, Y. Matsumoto, S. Mitsuoka, T. Kimura, K. Asai, T. Yana, T. Kawaguchi, K. Hirata, Phase II study of the modified weekly nab-paclitaxel regimen in previously treated patients with advanced non small cell lung cancer, American Journal of Clinical Oncology: Cancer Clinical Trials. 44 (2021) 613–618. https://doi.org/10.1097/COC.0000000000000876.

[35] S. Shoji, S. Miura, S. Watanabe, A. Ohtsubo, K. Nozaki, Y. Saida, K. Ichikawa, R. Kondo, T. Tanaka, K. Koyama, H. Tanaka, M. Okajima, T. Abe, T. Ota, T. Ishida, M. Makino, A. Iwashima, K. Sato, N. Matsumoto, H. Yoshizawa, T. Kikuchi, Phase II study of nanoparticle albumin-bound paclitaxel monotherapy for relapsed non-small cell lung cancer with patient-reported outcomes (NLCTG1302), Transl Lung Cancer Res. 11 (2022) 1359–1368. https://doi.org/10.21037/tlcr-22-89.
